# Silica NPs in PLA-Based Electrospun Nanofibrous Non-Woven Protective Fabrics with Dual Hydrophilicity/Hydrophobicity, Breathability, and Thermal Insulation Characteristics for Individuals with Disabilities

**DOI:** 10.3390/polym15204139

**Published:** 2023-10-18

**Authors:** Muhammad Omer Aijaz, Mohammad Rezaul Karim, Ibrahim A. Alnaser, Md Irfanul Haque Siddiqui, Abdulaziz K. Assaifan

**Affiliations:** 1 Center of Excellence for Research in Engineering Materials (CEREM), Deanship of Scientific Research (DSR), King Saud University, Riyadh 11421, Saudi Arabia; maijaz@ksu.edu.sa (M.O.A.); ialnaser@ksu.edu.sa (I.A.A.); 2 King Salman Center for Disability Research, Riyadh 11614, Saudi Arabia; msiddiqui2.c@ksu.edu.sa (M.I.H.S.);; 3 KACARE Research and Innovation Center, King Saud University, Riyadh 11421, Saudi Arabia; 4 Department of Mechanical Engineering, College of Engineering, King Saud University, Riyadh 11451, Saudi Arabia; 5 King Abdullah Institute for Nanotechnology, King Saud University, Riyadh 11451, Saudi Arabia; 6 Biomedical Technology Department, College of Applied Medical Sciences, King Saud University, Riyadh 12372, Saudi Arabia

**Keywords:** protective clothes, disability, nanoparticles, nanofibrous membrane, hydrophilicity, hydrophobicity, thermal insulation, breathability

## Abstract

A perfect protective fabric for handicapped individuals must be lightweight, waterproof, breathable, and able to absorb water. We present a multifunctional protective fabric in which one side is hydrophobic based on the intrinsic hydrophobic biopolymer polylactic acid (PLA) to keep the disabled person from getting wet, while the other side is super-hydrophilic due to embedded silica nanoparticles (NPs) to keep the disabled person safe from a sudden spill of water or other beverage on their skin or clothes. The porosity of the electrospun nanofibrous structure allows the fabric to be breathable, and the silica NPs play an important role as a perfect infrared reflector to keep the person’s clothing cool on warm days. Adding white NPs, such as silicon dioxide, onto or into the textile fibers is an effective method for producing thermally insulated materials. Due to their ability to efficiently block UV light, NPs in a network keep the body cool. Such a multifunctional fabric might be ideal for adult bibs and aprons, outdoor clothing, and other amenities for individuals with disabilities.

## 1. Introduction

Children, the elderly, and others with disabilities frequently struggle to move, eat, and dress themselves due to shaking or poor nerve coordination [1]. Food and drink spills can leave tough stains on garments and might irritate the skin. Therefore, it is essential to carefully select textiles for protective clothing, especially bibs or aprons used for feeding. An ideal protective fabric for disabled people must be lightweight, breathable, water-resistant, and air-permeable; it must allow liquid water absorption; and it must have strong thermal insulation capabilities [2,3,4].

The capacity of materials and apparel to breathe is a key factor in both indoor and outdoor clothing. Through the evaporation of moisture from many layers of clothing, a porous material allows for more heat loss. When clothing layers are impermeable, moisture is trapped between the skin and clothing, which causes heat to build up. This buildup of heat and moisture causes discomfort, wet skin, and skin abrasion. For people who are limited to a wheelchair or a bed, this problem is even worse. Nevertheless, liquid moisture may be removed from the skin via channels synthesized in fabrics made of fibrous material.

In hot temperatures, clothes and fabrics must have good thermal insulation qualities. Thermal insulation may be necessary depending on a person’s degree of activity and the outside temperature. It has been observed that two forms of UV radiation raise the possibility of skin cancer. The first kind of UV radiation is ultraviolet A (UVA), which has a wavelength of between 315 and 400 nm and is linked to skin aging. Ultraviolet B (UVB), the second kind of UV, is associated with skin burning and has a shorter wavelength (280–315 nm). In a hot climate where ultraviolet (UV) radiation can burn the skin when the temperature rises quickly, thermal insulation refers to the capacity to keep an individual cool. Because their activity level is so low, people with impairments have a hard time acquiring appropriate outdoor clothes. In addition, they must be able to dress appropriately when going outside and take off all additional layers when coming indoors. Protective clothing and accessories must be water-resistant to offer protection against liquids like rain, water, and other beverages. Waterproof garments and fabrics may be produced by altering the surface tension of textiles using coatings or layered nanofibrous membranes. A textile with several protective qualities should ideally have a porous structure to transmit air and water vapor and be hydrophobic to stop liquid water from accessing the membrane. Additionally, the membrane must be hydrophilic to promote the slipover of liquid on top of the fabric. There are several techniques that may be used to produce hydrophilic/phobic and breathable membranes, including melt-blowing, phase separation, template methods, fiber fibrillation, and electrospinning [5,6,7,8]. Due to the microporous nanofibrous structure produced through the aggregation of fibers, electrospinning has been shown to be a successful strategy for generating multifunctional protective clothing. By simply adjusting the fiber diameter, the membrane’s porosity structure can be adjusted, and it may be used as a protective fabric [5,9,10,11]. The degree of water resistance in apparel sold in stores is known to be inversely related to vapor permeability or breathability. The fabrication of waterproof-breathable fabric in the field of protective clothing can be achieved using electrospun nanofibers with different functional nanostructures. The electrospun web’s nano- or micropores offer good moisture and vapor release capabilities while maintaining water and wind resistance, making this material a promising candidate for use in waterproof-breathable textiles. Another benefit is its high thermal insulation due to the substantial amount of air in the nanofibrous pores. Adding white nanoparticles such as titanium oxide, zinc oxide, and silicon dioxide onto or into textile fibers is an effective method for producing thermally insulated materials. Typically, nanoparticles that form a network may effectively deflect UV radiation, keeping the body cool.

To date, significant efforts have been made in the fabrication of single-layer electrospun microporous membranes with waterproofness and breathability utilizing materials such as polyurethane, polyvinylidene fluoride, and polyacrylonitrile [12,13,14,15]. However, it is challenging to combine waterproofness with water-absorbing capabilities in protective clothing; nevertheless, several researchers have produced electrospun nanofibrous materials with dual-mode hydrophobic and hydrophilic qualities for protective clothing applications [16,17]. Mohsen et al. used a two-opposite-nozzle electrospinning setup to produce a bi-functional nanofibrous membrane made of polyurethane on one face and poly (2-acryloylamido-2-methylpropanesulfonic acid)-graphene oxide (PAMPS-GO) on the other face. The water vapor permeability of PAMPS nanofibers was examined, and it was discovered that the amount of graphene oxide improves the water vapor permeability. Similarly, Yuliang et al. prepared a dual layer of polyacrylonitrile and polystyrene with polydopamine coatings to study the behavior of liquid moisture transport through an electrospun membrane. The scientific community interested in membrane technology is now much more aware of the damaging consequences that petroleum-based polymers have on the earth and human health. Biodegradable polymers have recently attracted substantial interest as appealing substitutes for petroleum-based polymers due to their biocompatibility and environmental benefits [7,18]. One of the most promising and sustainable nanofibrous materials for use in protective clothing is PLA, a biopolymeric hydrophobic thermoplastic material with exceptional resilience, elasticity, and UV resistance. PLA-based electrospun nanofibers have attracted interest for use in medical implants, wound dressings [19], water treatment [20], scaffolds [21], drug delivery carriers [22], and fog collection applications [23] because of the extracellular matrix, large specific surface area, high porosity, small pore size, and suitable mechanical features.

Double-layered electrospun nanofibrous fabrics with a hydrophilic outside surface and a hydrophobic waterproof surface on the inside face were designed for the first time in this work using a sequential electrospinning setup. The pure PLA and modified PLA materials containing poly(ethylene glycol)-block-poly(propylene glycol)-block-poly(ethylene glycol) (PEG-PPG-PEG) and silica nanoparticles (SiO_2_) were utilized as polymers with hydrophobic and hydrophilic surfaces, respectively. Combining PEG-PPG-PEG and SiO_2_ with a PLA solution successfully increased the hydrophilicity of PLA electrospun membranes. PEG-PPG-PEG and SiO_2_-blended PLA membranes provided better water absorption and stability [20,23]. Therefore, PEG-PPG-PEG and SiO_2_ nanoparticles may be a viable option for improving the water stability of hydrophilic PLA membranes during prolonged aqueous media exposure as well as their breathability and heat-protecting characteristics. Field-emission scanning electron microscopy (FE-SEM) and contact angle (CA) tests were used to validate changes in the morphology and surface wettability of the hydrophobic and hydrophilic sides of the protective fabrics. Furthermore, tests for breathability, water stability, water transport rate (WTR), and thermal insulation capabilities were performed to investigate the protective behavior of both layers.

## 2. Materials and Methods

The main component of the nanofibrous fabrics was produced utilizing polylactic acid (PLA, LX175^®^, Filabot, Barre, VT, USA) and poly(ethylene glycol)-block-poly(propylene glycol)-block-poly(ethylene glycol) (PEG-PPG-PEG, Sigma Aldrich, Saint Louis, MO, USA) polymers. Silica nanoparticles (SiO_2_), a nanomaterial for embedding, of 10–20 nm were also obtained from Sigma Aldrich. The solvents dichloromethane (DCM) and dimethylformamide (DMF) purchased from Sigma Aldrich were used to produce an electrospinning solution. The hydrophilicity and hydrophobicity of the prepared fabrics were assessed using deionized water in this study.

### 2.1. Preparation of Hierarchically Structured Electrospun Fabrics

Hierarchically structured electrospun fabrics with dual hydrophilic and hydrophobic properties were prepared. The electrospinning solution for the hydrophobic layer consisted of PLA dissolved in a mixture of DCM and DMF at 50 °C for 1 h with a weight ratio of 4:1. Initially, a 10 *w*/*v*% PLA solution was prepared by first dissolving PLA in 12 mL of DCM at 50 °C for 1 h and then allowing the solution to cool down to room temperature. After cooling, 3 mL of DMF was added to the solution and stirred for 2 h. Electrospinning of the hydrophilic layer involved dissolving the 10 *w*/*v*% PLA and 2 *w*/*v*% PEG-PPG-PEG powders in a mixture of DCM and DMF at 50 °C for 1 h with a weight ratio of 4:1. To further increase the hydrophilicity, 260 mg (13 wt./wt.% of total PLA + PEG-PPG-PEG polymeric weight) of SiO_2_ nanoparticles were added to the prepared PLA/PEG-PPG-PEG solution. Prior to the electrospinning process, the PLA/PEG-PPG-PEG/SiO_2_ solution was kept for 24 h on a magnetic stirrer to ensure homogeneity and dispersion. 

**Scheme 1:**
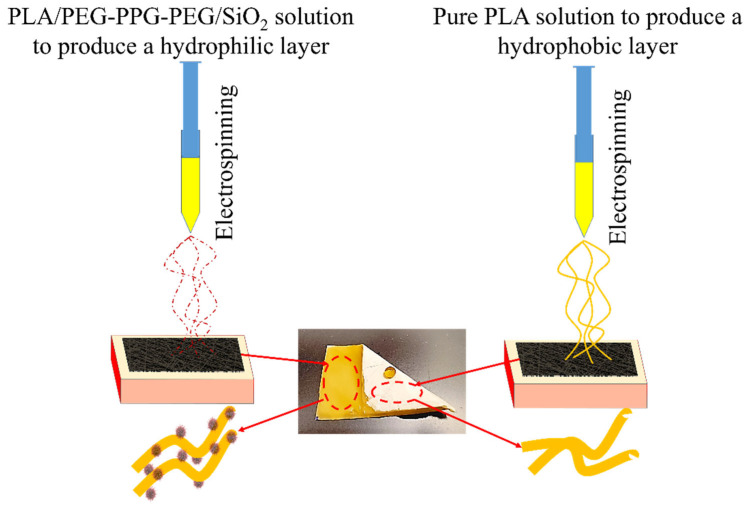
Fabrication of a double-layered nanofibrous non-woven fabric through a layer-by-layer electrospinning technique.

The doped pure PLA and the PLA/PEG-PPG-PEG/SiO_2_-blended solutions were placed into separate electrospinning syringes, and electrospinning was carried out in a stepwise manner as illustrated in Scheme 1. To begin electrospinning, the bottom layer of the fabric was first electrospun using a 100% PLA solution to generate the hydrophobic layer. Second, a syringe pump containing a blended solution of PLA/PEG-PPG-PEG/SiO_2_ was used to electrospun a super-hydrophilic layer over an existing electrospun hydrophobic PLA layer. The applied voltage was 18 kV throughout the electrospinning process, the flow rate was 0.8 mL/h, the humidity within the electrospinning chamber was 10%, and the collector distance was 15 cm. Electrospinning was stopped after the thickness of each layer reached ~100 ± 18 μm. Throughout the remainder of the text, the PLA/PEG-PPG-PEG/SiO_2_ membrane layer is referred to as the hydrophilic side, while the pure PLA membrane layer is referred to as the hydrophobic side of double-layered electrospun nanofibrous textiles.

### 2.2. Characterization

A field emission scanning electron microscope (FE-SEM: JSM-7600, JEOL, Tokyo, Japan) was used to examine the morphology of the produced electrospun textiles. Small portions of the fabric specimens were cut and fixed onto a stub using carbon tape before being placed into the FE-SEM apparatus for morphological investigation. A stub containing the specimen was then coated with platinum in order to enhance the sample’s electrical conductivity during the study. Later, coated samples were examined using FE-SEM in a high vacuum. The diameters of the nanofibers were measured using Adobe Photoshop CS6 software (v13.0.1) on roughly 50 randomly chosen nanofibers. Each layer’s thickness was measured using a probe and a thickness gauge (Ecotest Plus, Sheen Instruments, London, UK). The average membrane thickness for each sample was determined by averaging at least 10 measurements taken at different points for each layer.

**Scheme 2:**
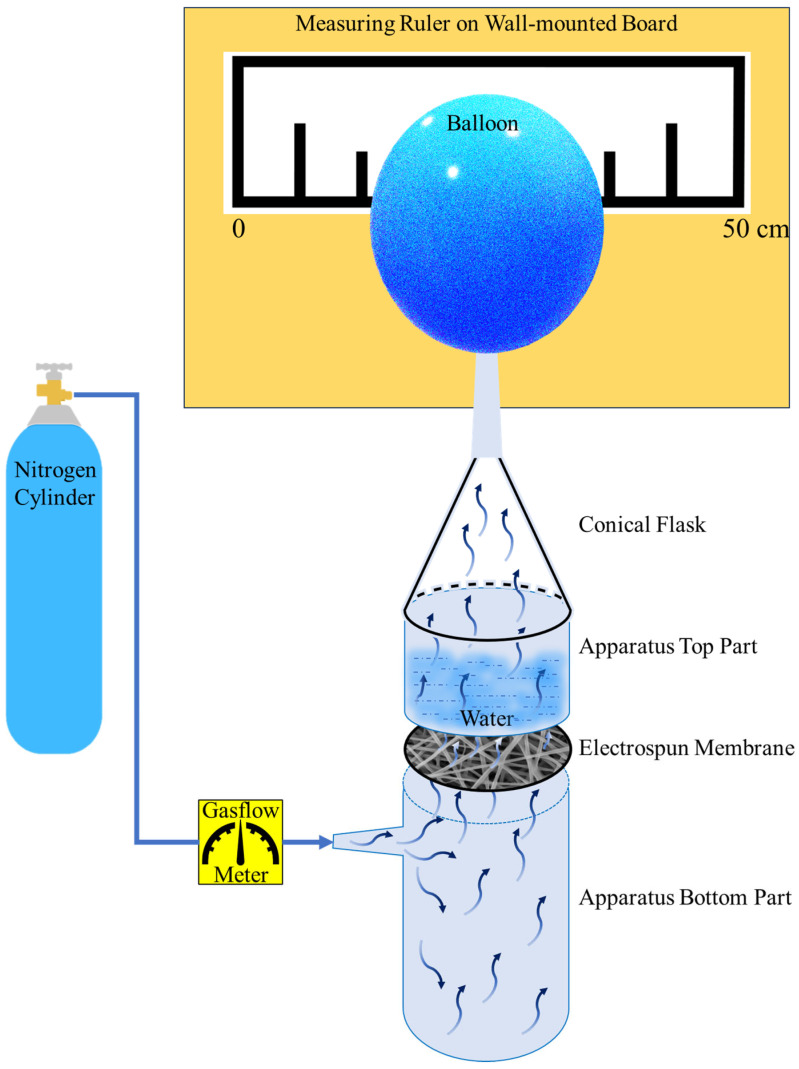
The breathability test setup.

The wettability behavior of the electrospun textiles was investigated using the CA goniometer (Model: OCA 15EC, Data Physics) apparatus. To calculate the droplet angle between the liquid and membrane surface, a droplet of deionized water was placed on the nanofibrous textiles. At least three CA values were averaged across multiple locations for each sample. After the electrospun textiles were successfully produced, the prepared PLA-based membranes’ breathability, aqueous stability, and WTR were determined. Scheme 2 shows a custom-designed breathability test setup that includes a conical flask, vacuum filtration apparatus, gas flow meter, ruler, large blue balloon, and nitrogen supply for calculating how easily air can move through the electrospun double-layered nanofibrous non-woven fabrics. 

To test the aqueous stability of the nanofibrous textiles, a small area of individual and combined layers of the fabrics were cut into squares, weighed (W*_I_*), and placed in a vial containing distilled water for 1–60 days at ambient temperature. Samples were taken out of the vial after the predetermined amount of time and dried for 24 h, and their dry weight (W*_F_*) was measured to evaluate weight loss. In order to test the fabrics’ capacity to be reused, each sample underwent five wet and dry cycles. Equation (1) was used to calculate the percentage of water stability [24]:

(1) $$Stability\;Percentage\left(\%\right)=\left(\frac{Dry\;Weight\;(W_{F})}{Initial\;Weight\;(W_{I})}\right)\times 100$$

The weight before and after full water absorption was used to calculate the WTR on the hydrophilic side of the manufactured electrospun fabrics. The fabric was cut into a rectangular shape (27.5 × 21 mm), which was then vertically positioned so that the bottom side of the fabric immediately touched the methyl orange solution to show the time-dependent movement of the water. This was performed to assess the WTR of the electrospun double-layered nanofibrous non-woven fabrics. Equation (2) was used to calculate the WTR once the fabric was totally submerged [23].

(2) $$WTR\;\left(\mathrm{mg}/{\mathrm{mm}}^{2}.\mathrm{hr}\right)=\left(\frac{\mathrm{Initial}\;\mathrm{Weight}\;(W_{I})-\mathrm{Final}\;\mathrm{Weight}\;\left(W_{F}\right)}{Area\;\left(A\right)\;.\;\;Time\;\left(t\right)}\right)$$

To test the heat barrier property of the electrospun fabrics, the reflection (%) and absorbance were calculated using UV-Vis spectroscopy. A UV-Vis-NIR scanning spectrophotometer (UV3600, Shimadzu, Kyoto, Japan) with an integrating sphere accessory for wavelengths between 200 nm and 800 nm was utilized to measure the optical responses of the manufactured materials. Later, to observe how the materials behaved thermally when exposed to sunlight, thermal infrared photographs of the double-layered electrospun nanofibrous fabrics with 15 × 18 cm dimensions were observed using an IR camera (FLIR E6). 

## 3. Results and Discussion

### 3.1. Preparation of the Electrospun Nanofibrous Fabrics

**Figure 1:**
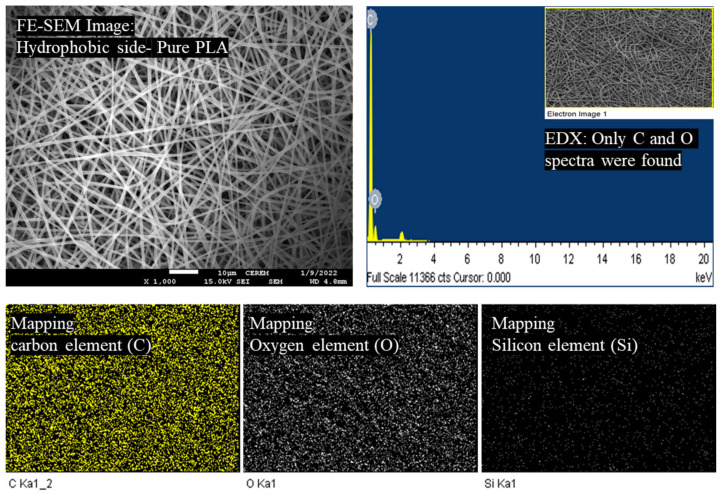
FE-SEM images and EDX results of the hydrophobic side of the electrospun nanofibrous fabrics with elemental mapping of carbon (C), oxygen (O), and silica (Si). The FE-SEM bar scale was fixed at 10 µm with 1000× magnification.

**Figure 2:**
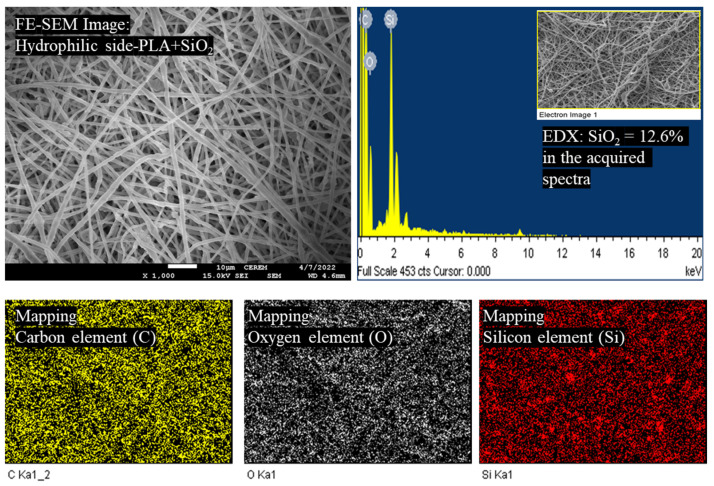
FE-SEM images and EDX results of the hydrophilic side of the electrospun nanofibrous fabrics with elemental mapping of carbon (C), oxygen (O), and silica (Si). The FE-SEM bar scale was fixed at 10 µm with 1000× magnification.

Figure 1 and Figure 2 display morphological EDX images of the multifunctional, hierarchically structured nanofibrous textiles and mapping evidence of silica nanoparticles. Figure 1 displays smooth, bead- and drop-free, fine PLA nanofibers with an average diameter of around ~960 nm [23]. Because the hydrophobic layer only contains pure PLA, EDX and mapping revealed the compositions of the basic polymeric materials. Figure 2 shows a morphological SEM image of the hydrophilic side with silica particles fused with fibers. The presence of silica in the nanofibers indicated in Figure 2 was confirmed with EDX data. Mapping photos of the manufactured nanofibrous textiles are shown in Figure 2, and the dark red shade in the elemental mapping of silica validates the presence of silica nanoparticles on the hydrophilic side of the fabrics [20].

### 3.2. Wettability Study 

**Figure 3:**
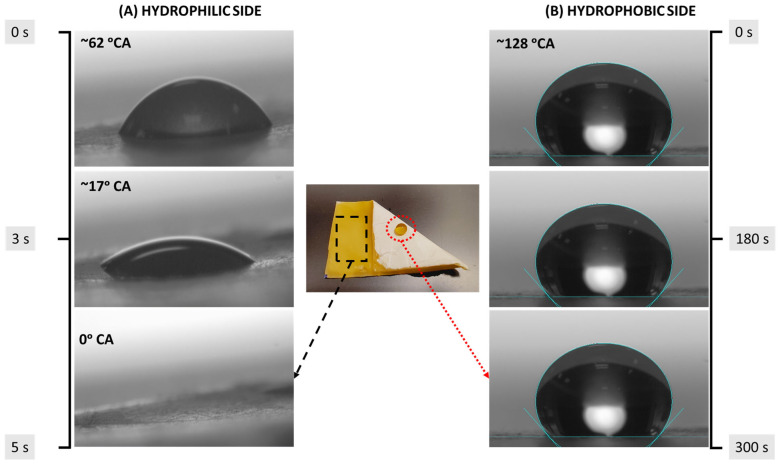
The contact angle of the electrospun double-layered nanofibrous non-woven fabrics. (**A**) Hydrophilic side after 5 s, and (**B**) hydrophobic side after 300 s at room temperature.

Contact angle measurements were taken on both sides of the prepared textile to confirm the dual nature of the electrospun textile. Initially, Figure 3A confirmed the hydrophilic nature of the top layer of the prepared fabrics, as the water drop surface angle quickly reached ~17 degrees within 3 s and the drop was fully absorbed within 6 s. This is because the addition of PEG and SiO_2_ to PLA confers hydrophilicity and attracts water molecules quickly [20]. This increase in hydrophilicity helps to protect the skin and clothes of a person from sudden spills while drinking beverages because the top hydrophilic layer of the protective fabric will absorb the spilled liquid, as shown in Figure 3A. On the other hand, the membranes’ hydrophobic nature was observed by flipping the textile surface from top to bottom and performing the CA test on the pure PLA side. Figure 3B shows the high and stable contact angle of ~128 degrees [23]. The shape of the water drop remained the same until the end of the test, as shown in Figure 3B. The hydrophobic nature confirmed the waterproofness of the electrospun textiles.

### 3.3. Breathability

**Figure 4:**
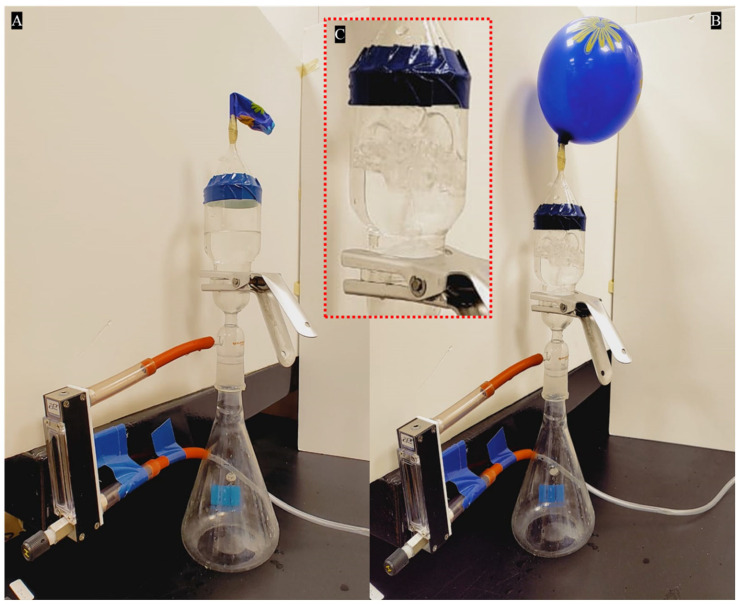
Breathability test images of the electrospun double-layered nanofibrous non-woven fabrics. (**A**) The balloon before nitrogen gas was supplied; (**B**) the balloon after opening the nitrogen gas supply; and (**C**) a zoomed-in image of the cell to show bubbles.

**Figure 5:**
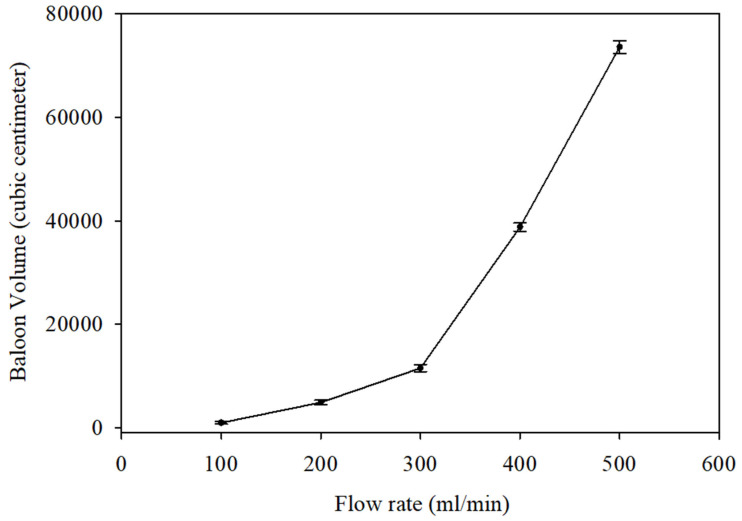
Effect of gas flow on the volume of the balloon gas collected to study the breathability of the electrospun double-layered nanofibrous non-woven fabrics.

The electrospun double-layered nanofibrous non-woven fabrics have SiO_2_ nanoparticles on one side of the fabric layer and a pure PLA layer on the other. SiO_2_ nanoparticles especially fill the gaps between nanofibrous structures; therefore, it is necessary to investigate the effect of the layers on breathability. To confirm waterproofness with air permeability (breathability), the fabrics were visualized and analyzed through a series of tests as shown in Figure 4A–C. As shown in Figure 4A, the blue balloon initially was flat with no gas; as soon as nitrogen gas was supplied (100 mL/h), the balloon became large quickly, demonstrating good breathability. To further verify the waterproofness and breathability of the membranes, the gas was passed at different gas flow rates from 100 to 500 mL/min. The effect of different gas flow rates on the volume of the balloon is shown in Figure 5, which confirms its robust waterproof property with good breathability.

### 3.4. Water Stability and WTR

**Figure 6:**
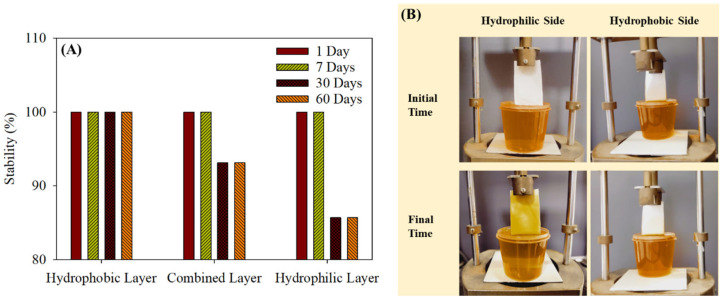
(**A**) Water stability and (**B**) WTR of the electrospun double-layered nanofibrous non-woven fabrics.

The water stability of the electrospun double-layered nanofibrous non-woven fabrics was evaluated, and the results are presented in Figure 6A. In the presence of water, the hydrophobic layer was perfectly stable. The stability of the dual layers and the hydrophilic layer was retained for up to 7 days, but after more than 30 days, the stability tended to recede to 93% and 80%, respectively. It was demonstrated that in the case of the double layers, deterioration began after 30 days and remained stable for 60 days.

The hydrophilicity of the obtained electrospun double-layered nanofibrous non-woven fabrics was further validated using WTR values, as shown in Figure 6B. An orange dye solution was directly applied to the produced membrane to monitor liquid movement and measure WTR. Figure 6B shows the initial and final water absorption. Because pure PLA is hydrophobic, the dye solution did not absorb water on the hydrophobic side of the clothing. However, it was found that the hydrophilic side quickly began absorbing water and that the color clearly changed on the hydrophilic side; the WTR value for the hydrophilic side was 20.7 mg·mm^−2^·h^−1^, whereas there was no WTR value found on the hydrophobic side of the nanofibrous clothing.

### 3.5. UV-VIS-NIR Reflection and IR Images

**Figure 7:**
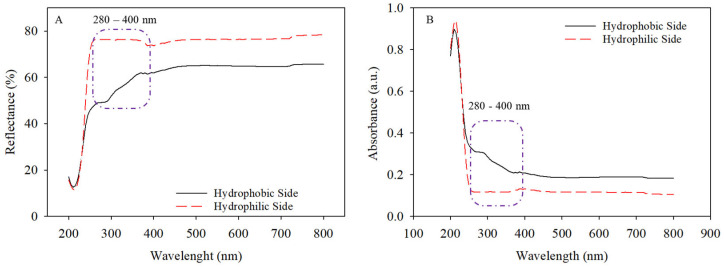
UV-Vis spectra of the electrospun double-layered nanofibrous non-woven fabrics. (**A**) reflection (%) and (**B**) absorbance curves.

To validate the UV reflection of the prepared electrospun fabrics, a UV-Vis test and IR image observation were performed. Figure 7A-B illustrates the reflection of the different layers of the system for wavelengths between 200 and 800 nm. The percentage blockings of UV-A (315–400 nm) and UV-B (280–315 nm) regions were 50 and 60% for the pure PLA fabric (hydrophobic side) and 78 and 74% for the double-layered fabrics. The hydrophilic layer with SiO_2_ nanoparticles showed efficient blocking of UV radiation in both regions. The average reflectance in the visible wavelength region (400–800 nm) of the pristine PLA fabrics was 62%, but it increased to 78% after adding SiO_2_ nanoparticles. In terms of reflectance (%), the hydrophilic side of the fabrics with SiO_2_ outperformed by ~28%. However, the layer without SiO_2_ nanoparticles showed less reflectance. The electrospun double-layered nanofibrous fabric almost entirely blocked out UV radiation, as shown by the absorbance curve in Figure 7B, which further supported the findings that the nanofibrous fabrics had enhanced heat resistance when exposed to sunshine [25,26].

**Figure 8:**
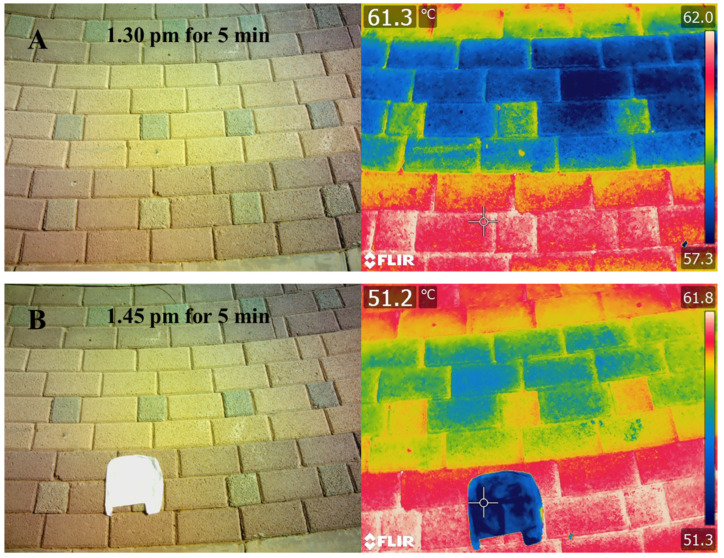
Digital and infrared images of a (**A**) brick floor in bright sunlight and the (**B**) brick floor covered with the electrospun double-layered nanofibrous non-woven fabric in bright sunlight.

**Figure 9:**
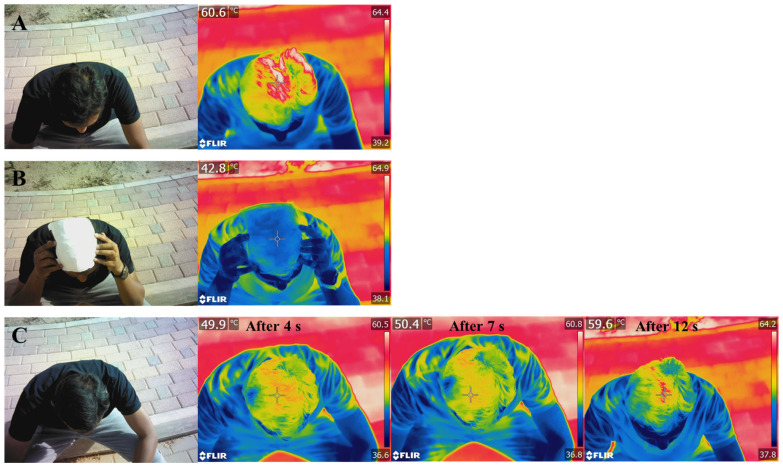
Digital and infrared images of (**A**) a human head in direct bright sunlight, (**B**) human head in direct sunlight while wearing the electrospun protective clothing, and (**C**) human head in bright sunlight just after removing the electrospun double-layered nanofibrous non-woven fabric.

Additionally, images from an infrared camera were taken to investigate the thermal effects of textiles under direct sunlight. Figure 8 and Figure 9 show digital and infrared images of a brick floor and a human head with and without protective clothing, respectively. The temperature on the brick ground in Figure 8A was measured at ~61 °C; however, in Figure 8B, using the same floor, the protective clothes were subjected to a significantly lower temperature of ~51 °C. Similarly, Figure 9A–C displays digital and infrared images of a human head with and without protective clothing. In Figure 9A,B, the temperature of the head was recorded at ~60 °C, whereas the temperature of the head was ~42 °C when protective apparel was worn. When the protective clothing was worn, the head became 40% cooler, which is a significant decrease in temperature in the Middle East region, particularly during the summer. Digital and infrared images were used to validate the temperature of the head. Figure 9C demonstrated that even after 4 s of the fabric being removed, the head’s temperature remained stable at 30% cooler levels before falling back to ambient temperature in under 14 s.

## 4. Conclusions

In summary, utilizing the stepwise electrospinning technique, we were able to fabricate protective clothing with a hydrophobic pure PLA layer on one surface and a super-hydrophilic PLA/PEG-PPG-PEG/SiO_2_ layer on the other. The protective clothing preserves the textile’s excellent breathability and flexibility while also possessing other required qualities, including waterproofness, high water stability, IR reflection, and the capacity to swiftly absorb spilled liquids. The electrospun nanofibrous structure containing SiO_2_ nanoparticles plays a crucial role in boosting IR reflection effectiveness while maintaining excellent breathability. The electrospun double-layered nanofibrous fabric almost entirely blocked UV radiation, which supported the findings that nanofibrous fabric has enhanced heat resistance when exposed to sunshine. The breathability test revealed that the nanofiber membranes could fix and test the air permeability. In the presence of water, the hydrophobic layer was perfectly stable. The stability of the dual layers and the hydrophilic layer was retained for up to 7 days, but after more than 30 days, the stability tended to recede to 93% and 80%, respectively. It was demonstrated that in the case of the double layers, deterioration began after 30 days and remained stable for 60 days. Moreover, the membranes managed to reduce heat by ~40%, which is a significant decrease in temperature in the Middle East area, particularly during the summer. This biopolymeric-based protective clothing is fabricated from renewable resources and is environmentally friendly. It may be used to make outdoor coverings, kid- and adult-sized protective bibs and aprons, protective clothing, and novel accessibility features when feeding individuals with disabilities.

## Data Availability

Not applicable.
